# Insulin and 5-Aminoimidazole-4-Carboxamide Ribonucleotide (AICAR) Differentially Regulate the Skeletal Muscle Cell Secretome

**DOI:** 10.3390/proteomes9030037

**Published:** 2021-08-03

**Authors:** Alba Gonzalez-Franquesa, Lone Peijs, Daniel T. Cervone, Ceren Koçana, Juleen R. Zierath, Atul S. Deshmukh

**Affiliations:** 1 Novo Nordisk Foundation Center for Basic Metabolic Research, University of Copenhagen, 2200 Copenhagen, Denmark; albagf@sund.ku.dk (A.G.-F.); peijs@sund.ku.dk (L.P.); daniel.cervone@sund.ku.dk (D.T.C.); ceren.kocana@gmail.com (C.K.); Juleen.zierath@ki.se (J.R.Z.); 2 Integrative Physiology, Department of Molecular Medicine and Surgery, Karolinska Institutet, 17177 Stockholm, Sweden; 3 Clinical Proteomics, Novo Nordisk Foundation Center for Protein Research, University of Copenhagen, 2200 Copenhagen, Denmark

**Keywords:** secretomics, skeletal muscle, metabolism, insulin, AMPK

## Abstract

Skeletal muscle is a major contributor to whole-body glucose homeostasis and is an important endocrine organ. To date, few studies have undertaken the large-scale identification of skeletal muscle-derived secreted proteins (myokines), particularly in response to stimuli that activate pathways governing energy metabolism in health and disease. Whereas the AMP-activated protein kinase (AMPK) and insulin-signaling pathways have received notable attention for their ability to independently regulate skeletal muscle substrate metabolism, little work has examined their ability to re-pattern the secretome. The present study coupled the use of high-resolution MS-based proteomics and bioinformatics analysis of conditioned media derived from 5-aminoimidazole-4-carboxamide ribonucleotide (AICAR—an AMPK activator)- and insulin-treated differentiated C2C12 myotubes. We quantified 858 secreted proteins, including cytokines and growth factors such as fibroblast growth factor-21 (Fgf21). We identified 377 and 118 proteins that were significantly altered by insulin and AICAR treatment, respectively. Notably, the family of insulin growth factor binding-proteins (Igfbp) was differentially regulated by each treatment. Insulin- but not AICAR-induced conditioned media increased the mitochondrial respiratory capacity of myotubes, potentially via secreted factors. These findings may serve as an important resource to elucidate secondary metabolic effects of insulin and AICAR stimulation in skeletal muscle.

## 1. Introduction

Skeletal muscle is an important tissue in the maintenance of postprandial glucose homeostasis [1] and a major site for insulin resistance in metabolic disease [2]. In skeletal muscle from people with a normal glucose tolerance, insulin effectively stimulates glucose transporter 4 (GLUT4) translocation to the sarcolemmal membrane to increase glucose uptake. Exercise is an effective therapeutic intervention to mitigate metabolic disease, partly owing to its ability to enhance cellular glucose uptake via insulin-independent pathways. Exercise and AMPK-activators (e.g., AICAR) that simulate energetic stress can coordinate protein signaling events that lead not only to increases in glucose transport and catabolic ATP-synthetic pathways, but also the transcriptional upregulation of genes [3,4]. Skeletal muscle responds to a variety of stimuli by altering genes that encode proteins destined for secretion into systemic circulation [5]. There is now growing appreciation that skeletal muscle acts as an endocrine organ through the secretion of proteins/peptides, termed ‘myokines’ [6]. While the understanding of the skeletal muscle secretome remains incomplete, myokines (e.g., interleukin-6; IL-6) are proven regulators of inflammation, immune function and energy metabolism [7,8]. Insulin- and AMPK-dependent pathways are major regulators of skeletal muscle metabolism. Thus, understanding how these pathways govern skeletal muscle protein secretion may provide insights into their complex regulation of whole-body metabolism and inter-organ communications.

Recent developments in mass spectrometry (MS)-based proteomics have led to more robust analysis of the plasma proteome [9]. However, detecting low abundant secreted proteins remains challenging due to the high dynamic range of plasma [10]. Although investigating cellular conditioned media has become an alternative strategy, cellular secretomics presents its own obstacles, including serum and media contamination, cellular damage, and in some cases, disentangling the effects of treatment from the serum-starvation of cells [11,12]. To date, the quantitative coverage of the secretome of the immortalized mouse myoblast C2C12 cell line is variable, and detailed comparisons of its composition following the activation of distinct signaling pathways are underexplored [13,14,15]. This is surprising given that differentiated C2C12 myotubes are widely used to garner mechanistic insight into skeletal muscle metabolism [16]. Previous studies have focused on secretome analysis of undifferentiated myoblasts [17], or myotubes treated with electrical pulse- or cytokine-stimulation [18,19]. Here, we examined the secretome of differentiated C2C12 myotubes following stimuli that are consistently applied in metabolic experiments to activate signaling pathways that underpin metabolic homeostasis. Specifically, we mapped the secretome using high-resolution tandem mass spectrometry (LCMSMS)-based proteomics analysis of conditioned media and cell lysate following six hours of either AICAR- or insulin-stimulation. Given that AICAR and insulin initiate different signaling pathways, we hypothesized that the composition and dynamics of myokine secretion would be distinct.

## 2. Materials and Methods

### 2.1. Cell Culture and Treatment of C2C12 Myotubes

C2C12 myoblasts were grown in 6-well plates in DMEM (Gibco—ThermoFisher Scientific: Waltham, MA, USA) supplemented with 10% fetal bovine serum (FBS; Sigma-Aldrich: St. Louis, MO, USA) and antibiotics (1% Penicillin-Streptomycin, Gibco) in gassed and humidified air (5% CO_2_). Myoblasts were grown until confluence in growth media. Differentiation was induced with the addition of DMEM supplemented with a 2% horse Serum (Sigma-Aldrich) and antibiotics. All experiments were performed in myotube cultures after 5 days of differentiation. Differentiated myotubes were washed five times with conditioned media (high-glucose, serum- and phenol red-free DMEM supplemented with 4 g/L L-glutamine and 1% antibiotics mix). These washes were performed to reduce any likelihood of serum contamination in the media for proteomics analysis. Myotubes were treated for 6 h with or without 0.5 mM AICAR (A611700, Toronto Research Chemicals: North York, ON, Canada) or 10 nM insulin (I9278, Sigma-Aldrich). The conditioned media was then collected and myotubes were lysed with protein lysis buffer (10% glycerol, 1% IGEPAL, 150 mM NaCl, 50 mM HEPES, 20 mM beta-glycerophosphate, 10 mM NaF, 1 mM EDTA, 1 mM EGTA, 1 mM Na-butyrate, 20 mM Na-pyrophosphate, 1X SigmaFast Protease Inhibitor) for cellular proteome analysis.

### 2.2. Sample Preparation for Secretome Analysis

Following 6 h of treatment, the conditioned media was collected, subjected to centrifugation (8000× *g*, 10 min), and filtered (0.22 µm filters) to remove cellular debris. Urea (Sigma-Aldrich) was added to 900 µL of conditioned media to yield a final concentration of 2 M, and 90 µL of Tris HCl (1 M, pH 8.5) was added to ensure proper pH for enzymatic digestion. Sample preparation proceeded using a modified version of the filter-aided sample preparation (FASP) protocol [20]. Briefly, samples were incubated at 56 °C for 10 min, prior to centrifugation (8000× *g*, 10 min), and the supernatant was transferred to a centrifugal filter unit (Microcon-30kDa, Millipore: Burlington, MA, USA), spun down, and washed two times with urea buffer (2 M urea in 0.1 M Tris HCl, pH 8.5). DTT (10 µmol) and IAA (5.5 µmol) in urea buffer were added for reduction and 30 min alkylation in the dark, respectively. Proteins were digested with LysC (1 µg) for 3 h at 37 °C, and trypsin (1 µg) was then added for overnight digestion. Peptides were eluted with an additional elution step with H_2_O ensured optimal peptide recovery. The samples were acidified with 100% trifluoroacetic acid to halt enzymatic digestion. Peptides were then desalted on reverse-phase C18 StageTips [21] and eluted in two steps (1) 40% acetonitrile in 0.5% acetic acid and (2) 60% acetonitrile in 0.5% acetic acid. The organic solvents were evaporated at 45 °C in a sample concentrator and peptides were re-suspended in 2% acetonitrile in 0.1% formic acid.

### 2.3. Sample Preparation for Cellular Proteome Analysis

The cellular proteome was examined in cell lysate with 6 replicates per group. Proteins were acetone-precipitated (with 4 volumes of ice-cold acetone), vortexed and left overnight at −20 °C. Samples were subjected to centrifugation to generate a pellet and then washed with 100% acetone, twice with 80% acetone, and left to dry. The pellet was resuspended with a urea/thiourea (U/T) buffer (6 M/2 M, respectively). Proteins (20 µg) underwent 1 h digestion with LysC (2.5 µg) at 37 °C. The sample was then diluted 1:4 with 25 mM Tris (pH 8.5) before overnight digestion with Trypsin (2.5 µg). The enzymatic reaction was terminated, and the peptides were desalted as described above.

### 2.4. LCMS/MS Analysis

LCMS instrumentation consisted of an Easy Nanoflow UHPLC coupled via a nano-electrospray ion source to a QExactive mass spectrometer-HFX (ThermoFisher Scientific). Peptides were separated on a 50 cm column with 75 µm inner diameter packed in-house with ReproSil-Pur C18-aq 1.9 µm resin (Dr. Maisch: Ammerbuch-Entringen, Germany). For secretome analysis of conditioned media, peptides were loaded in a 0.5% formic acid buffer and eluted with a 140 min linear gradient with acetonitrile (80%). Mass spectra were acquired in a data-dependent manner (Full MS/dd-MS^2^ Top 12 scan mode), with the following parameters for full MS: mass range 300–1750 *m*/*z*, resolution 60,000 at *m*/*z* 200, AGC target 3e6, maximum injection time of 45 ms; and for dd-MS^2^: resolution 60,000 at *m*/*z* 200, AGC target 1e5, maximum injection time of 120 ms, and NCE of 28%. For the cellular proteome analysis, peptides were loaded in a 0.5% formic acid buffer and eluted with a 100 min linear gradient with acetonitrile (98%). Mass spectra were acquired in a data-dependent manner (full MS/dd-MS^2^ Top 15 scan mode), with the following parameters for full MS: mass range 300–1650 *m*/*z*, resolution 60,000 at *m*/*z* 200, AGC target 3e6, maximum injection time of 25 ms; and for dd-MS^2^: resolution 60,000 at *m*/*z* 200, AGC target 1e5, maximum injection time of 25 ms, and NCE of 27%. Data were acquired using Xcalibur software.

### 2.5. Computational LCMS/MS Data Analysis

Mass spectra were analyzed using MaxQuant (v1.5.3.30) and the built-in label-free quantification algorithm was used for protein quantification [22]. The initial maximum tolerance for mass deviation was set to 6 ppm for monoisotopic precursor ions and 20 ppm for MS/MS peaks. Enzyme specificity was set to trypsin, defined as a C-terminal to arginine and lysine (excluding proline). A maximum of two missed cleavages was permitted and a minimum peptide length of seven amino acids was required. Carbamidomethyl cysteine was set as a fixed modification, while *N*-terminal acetylation and methionine oxidation were set as variable modifications. The spectra were queried in the Andromeda search engine against the mouse UniProt sequence database, 248 common contaminants and the reverse of all sequences. The MS/MS spectra were searched against the mouse UniProt FASTA database (version November 2017). The false discovery rate (FDR) for protein/peptide identification was set to 1%. To match identifications across different runs, the “match between runs” option in MaxQuant was enabled with a retention time window of 30 s. In the case of identified peptides that were shared between two or more proteins, these were combined and reported in the protein group. Contaminants and reverse identifications were removed from further data analysis. Protein quantification was performed based on razor and unique peptides. The number of razor and unique peptides for each quantified protein are indicated in Table S1. Proteins in the conditioned media and cell lysate were only analyzed if they were quantified in all samples (conditioned media: *n* = 4–5/group; proteome: *n* = 6/group). One control sample was removed as a technical outlier, likely due to unknown contaminants. For missing values, data were imputed using a downshifted (1.8 standard deviation from the mean) Gaussian distribution with random numbers having 30% of the true standard deviation of the valid experimental values. This simulates the distribution of low signal values. Gene Ontology (GO) biological process (GOBP), molecular function (GOMF), cellular component (GOCC) and UniProt Keywords were used to assign categorical annotations to identified proteins. The Pfam database (pfam.xfam.org (accessed 1 June 2015) was used for domain predictions and enrichment analyses.

### 2.6. Immunoblotting and ELISA

Criterion XT Bis-Tris Gels (4–12% polyacrylamide, BioRad: Hercules, CA, USA), XT MES Buffer (BioRad) and Trans-Blot^®^ Turbo™ Midi Transfer Packs were used for electrophoresis and immunoblotting (*n* = 2–4) on PVDF membranes. Phospho-AMPKα (Thr^172^; 2531) and Phospho-AKT (Ser^473^; 4060) antibodies were sourced from Cell Signaling Technology: Danvers, MA, USA. Images were obtained with a ChemiDoc + XRS and immunoblots were quantified with ImageLab analysis software (BioRad). IL-6 was quantified in the conditioned media using a commercially available mouse IL-6 ELISA kit (*n* = 10; 431301, BioLegend: San Diego, CA, USA).

### 2.7. Mitochondrial Respiration

Mitochondrial respiration was measured in C2C12 myotubes (*n* = 27–30 from two independent experiments) after 5 days of differentiation using a Seahorse XFe96 Analyzer (Agilent, Santa Clara, CA, USA). Following 4 h of cell treatment with either conditioned media (mixed 1:1 with differentiation media) or differentiation media supplemented with either insulin (5 nM) or AICAR (0.25 mM), the MitoStressTest protocol was performed according to the manufacturer’s instructions. Briefly, the instrument monitored oxygen concentration and performed the following titrations: (1) 1 µM oligomycin, (2) 2 µM FCCP, (3) 2 µM rotenone + 2 µM antimycin-A (Sigma). Data were considered as outliers if they were greater than 2.5 times the median absolute deviation. Basal respiration, proton leak, ATP production, maximal respiration and spare capacity were calculated based on values of oxygen consumption rate (OCR).

## 3. Statistics

All statistical, principal component and enrichment analyses of the MaxQuant output were performed using Perseus (version 1.6.15.1) [23]. For the comparative analysis of the insulin- and AICAR-induced secretomes, the experimental conditions were compared (Ctrl vs. AICAR and Ctrl vs. insulin) using a Student’s *t*-test with a Benjamini–Hochberg correction (FDR = 5% and S0 = 0.1). Enrichment analyses were performed using a Fisher’s exact test (threshold value: 0.02) on differentially regulated secreted proteins (compared to the full secretome), or using 2D enrichment analyses [24]. Immunoassay and mitochondrial respiration data were analyzed using a one- and two-way ANOVA, respectively, with a Holm–Šídák post hoc test for multiple comparisons. Specific statistical tests, thresholds for significance and sample size are indicated in figure legends.

## 4. Results and Discussion

### 4.1. Validating the Cellular Effects of Insulin and AICAR

**Figure 1:**
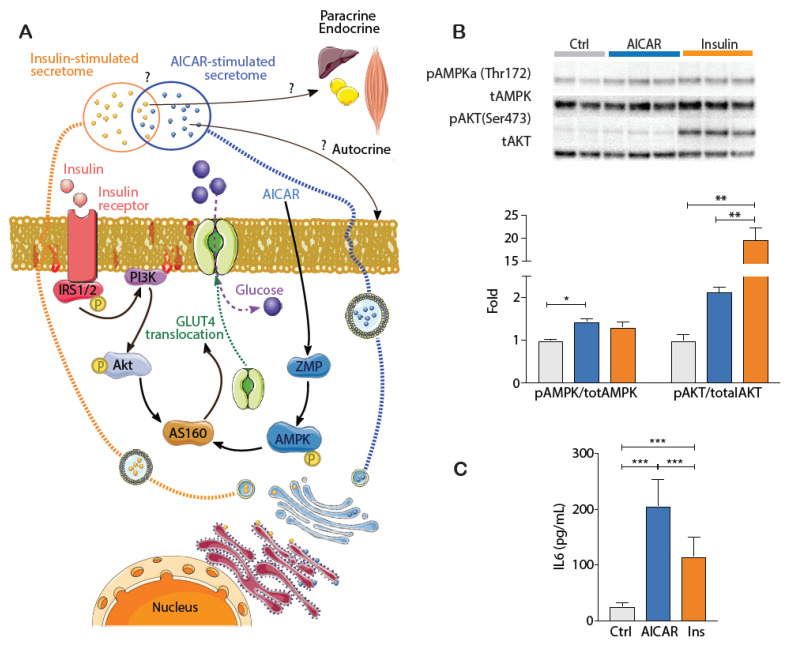
(**A**): Schematic depicting the pharmacological activation of distinct cellular signaling pathways in skeletal muscle induced by insulin (10 nM) and AICAR (0.5 mM), which elicit changes to cellular metabolism (e.g., glucose transport shown), as well as the secretion of proteins that may have auto-, para- or endocrine effects. (**B**): Immunoblot analysis (*n* = 2–4) of the downstream phosphorylation of regulatory proteins AKT (Ser^473^) and AMPK (Thr^172^). (**C**): IL-6 concentration in conditioned media of C2C12 myotubes following vehicle (control), AICAR or insulin administration (*n* = 10). Data wereanalyzed using a one-way ANOVA with a Holm-Šídák *post hoc* test. * *p* < 0.05; ** *p* < 0.01; *** *p* < 0.0001.

AICAR and insulin activate distinct cellular pathways that exert widespread effects on metabolism (Figure 1A). Prior to LCMS/MS experiments identifying secreted proteins, the cellular signaling effects of both AICAR and insulin were assessed. As depicted, AICAR is transported by adenosine transporters and can undergo subsequent intracellular conversion to ZMP (Figure 1A). This AMP-mimetic potently activates AMPK. Accordingly, AICAR treatment increased AMPK activation as evidenced by the increased phosphorylation at its Thr^172^ residue (Figure 1B) [25]. In contrast, insulin initiates its cellular effects by binding the insulin receptor (IR) on the cell surface. Several subsequent signaling events lead to downstream phosphorylation of the key intermediary protein kinase AKT, to upregulate its activity [26]. The robust increase in AKT phosphorylation at Ser^473^ confirmed the cellular action of insulin in myotubes (Figure 1B). To determine whether myotubes were actively secreting low abundant proteins, we measured the secretion of IL-6 into conditioned media. IL-6 is a well-known exercise-induced myokine that has also generated interest through the regulation of peripheral metabolic, inflammatory and immune processes [27,28]. IL-6 concentration in the conditioned media was increased with both treatments, albeit the increase was expectedly greater in response to AICAR stimulation (Figure 1C). For the remaining analyses, we sought to generate an extensive catalogue of myokines with potential roles in metabolism, and examined whether they are differentially regulated by insulin and/or AICAR.

### 4.2. Secretomics Analysis and Filter for Secreted Proteins

**Figure 2:**
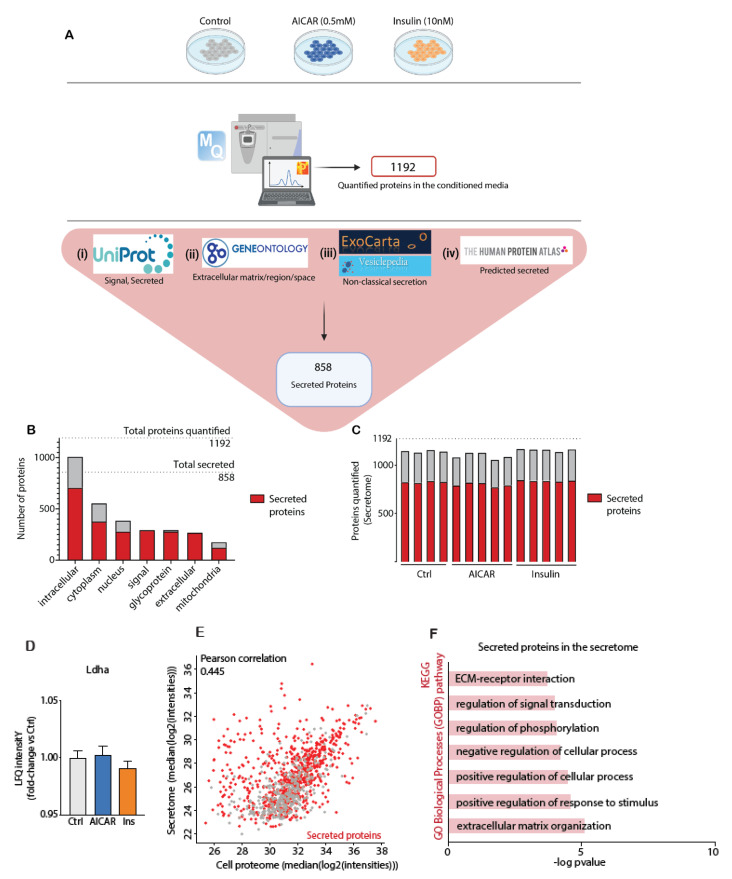
(**A**): Schematic describing the workflow used to filter for secreted proteins in the conditioned media. (**B**): Annotations of the proteins in the conditioned media (UniProt keywords: signal, glycoprotein; GOCC: intracellular, cytoplasm, nucleus, extracellular mitochondria). Secreted proteins are highlighted in red. (**C**): Total quantified proteins in the conditioned media across each treatment (Ctrl, AICAR, insulin), with secreted proteins shown in red. (**D**): Proteomic analysis of the conditioned media provides the relative abundance (fold-change) of lactate dehydrogenase A (LDHA; a marker of cellular damage), with each treatment. (**E**): Scatter plot and Pearson correlation analysis of the cellular and conditioned media proteomes. (**F**): KEGG pathways and Gene Ontology Biological Processes (GOBP) enriched by secreted proteins quantified in the conditioned media with each treatment.

Raw MS files from the AICAR and insulin-treated conditioned media and cell lysate samples were processed in MaxQuant software where proteins were quantified using the built-in label-free algorithm [29]. We quantified 1192 and 3912 proteins in the C2C12 secretome (conditioned media) and cellular proteome, respectively (Tables S1 and S2). We next applied a systematic bioinformatics approach to predict potential secreted proteins in the conditioned media. Protein secretion is complex and occurs through several distinct pathways [30]. Classically secreted proteins are targeted for translocation through the endoplasmic reticulum (ER) membrane by an N-terminal signal peptide sequence. However, the secretome also comprises proteins released via non-classical secretory pathways [31,32]. Annotating non-classically secreted proteins is a key challenge in the field of secretomics and it relies on the continual evolution of bioinformatics databases. The growing interest in identifying non-classically secreted proteins has led to the curation of two databases, Vesiclepedia [33] and Exocarta [34], which contain proteins known to be secreted in microvesicles. Therefore, to filter for potential secreted proteins, we leveraged the combined use of (i) Uniprot keywords that identify proteins annotated as secreted or that contain a signal peptide, (ii) GOCC annotations that identify proteins located in the extracellular compartment (i.e., extracellular matrix, region or space), (iii) the Human Protein Atlas list of predicted secreted proteins [35], and (iv) the Vesiclepedia and Exocarta databases that identify proteins found in microvesicles (Figure 2A). Our analysis led to the identification of 858 potential secreted proteins, henceforth known as “secreted proteins”, which were annotated to multiple protein categories (Figure 2A,B). These 858 proteins constituted 72% of proteins quantified in the conditioned media.

The secreted proteins (Figure 2B) were annotated within several cellular compartments: intracellular (705), cytoplasm (366), nucleus (274), mitochondria (116) and extracellular matrix/region/space (261). There was no clear difference in the total number of quantified proteins or secreted proteins in the secretome with each treatment (Figure 2C). As expected, many of the secreted proteins also contained a signal peptide (290) or were glycoproteins (277). While the high abundance of intracellular proteins might initially be explained by potential apoptosis following serum-deprivation of cells, proteomic analysis revealed that cytosolic protein lactate dehydrogenase A (LDHA), a marker of membrane leakage/damage, was unchanged in the conditioned media from cultured myotubes (Figure 2D). This suggests that any relative contribution of cell death/lysis to the observed differences in secreted protein responses across experimental groups is unlikely. Further, although some degree of apoptosis can be expected with serum deprivation of cells, it is also conceivable that these intracellular proteins have multiple biological roles within different cellular compartments (i.e., both intra- and extracellularly), known as protein moonlighting [36]. Ninety percent of proteins (1065) in the media were also detected in the cellular proteome (Figure 2E). However, a weak correlation (Pearson = 0.445; R^2^ = 0.198) was observed when comparing the median LFQ intensities of proteins from both datasets (Figure 2E). In this correlation, secreted proteins generally displayed higher intensities in the secretome, further validating the application of these filters in identifying bona fide secreted proteins (Figure 2E, secreted proteins in red).

To date, though varied in proteomics methodology and MS-based instrumentation, several studies have examined the C2C12 secretome [14]. In most cases, the depth of coverage in conditioned media ranges from fewer than 100 proteins [15,37] to upwards of 700 proteins [17]. To our knowledge, the highest coverage of the C2C12 secretome stems from previous work from our own group. The past study identified >4000 proteins in the conditioned media and subsequent filtering led to the quantification of 1073 secreted proteins in C2C12 conditioned media following prolonged (16 h) palmitate treatment [11]. The high palmitate concentration (0.5 mM), 16 h of serum-starvation and longer LC gradient (270 min) may have collectively contributed to an increase in protein identifications [11]. To our knowledge, past studies have not comparatively examined the Vesiclepedia or Exocarta databases, perhaps since these resources rely on the reporting of proteins identified in isolated/purified microvesicles, which can be fraught with cellular contaminants/debris [38]. Accordingly, there is currently no gold-standard method to harvest microvesicles and quantify their cargo [38]. Regardless of whether limitations to predicting secreted proteins stem from databases or experimental setup, the candidate secreted proteins require validation. In the present work, nine known cytokines (e.g., Spp1, Cx3cl1, Grem1, Csf1) and several growth factors and matrix metalloproteinases were detected in the secretome (Table S1). Among them were several secreted proteins including the bone morphogenic protein 1 (Bmp1), growth factors such as transforming growth factor beta (Tgf-β1-3) and Fgf21, and the proteases matrix metalloproteinase 2 (Mmp2) and dipeptidyl-peptidase 3 (Dpp3) (Table S1). These proteins exert diverse metabolic functions i.e., Bmp1 is vital for the formation and development of the extracellular matrix [39] and its antagonist, gremlin-1 (Grem1), was also quantified in the conditioned media [40]. Recent work has provided evidence that Grem1 is predominately expressed in skeletal muscle satellite cells, and the abundance of this protein is reduced in the skeletal muscle of humans with obesity [40]. Nevertheless, the role of Grem1 as a myokine remains largely unexplored. In addition, Fgf21 has emerged as a regulator of substrate metabolism [41], whereas Dpp3 is known to coordinate oxidative stress, inflammation and apoptosis [42].

Of the 294 glycoproteins quantified in the conditioned media, 279 (95%) were secreted proteins (Figure 2B). This reinforces the importance of using multiple annotation terms in secretomics, as many glycoproteins, which may be truly secreted, are not yet annotated as such [43]. We further performed a comprehensive analysis of biological processes enriched in secreted proteins (Figure 2F). Our data provides evidence for the enrichment of several categories including signal transduction, phosphorylation, and ECM regulation (e.g., receptor interactions and matrix organization). This supports the notion that these secreted proteins may impart diverse effects on cellular metabolism. Only a few previous studies have examined the insulin- and AICAR-stimulated secretomes of skeletal muscle cells [15,44]. These studies were performed in L6 myotubes and only identified 153 and 74 proteins in the secretome, respectively. As such, the present work is the first to extensively characterize the skeletal muscle cell secretome in response to insulin and AICAR, which are frequently used to perturb signaling pathways controlling cellular metabolism. Other recent work also supports the use of myotubes as a model to characterize the secretome in response to nutritional stressors, such as following changes to amino acid provision [45].

### 4.3. AICAR and Insulin Differentially Regulate Muscle Secreted Proteins

**Figure 3:**
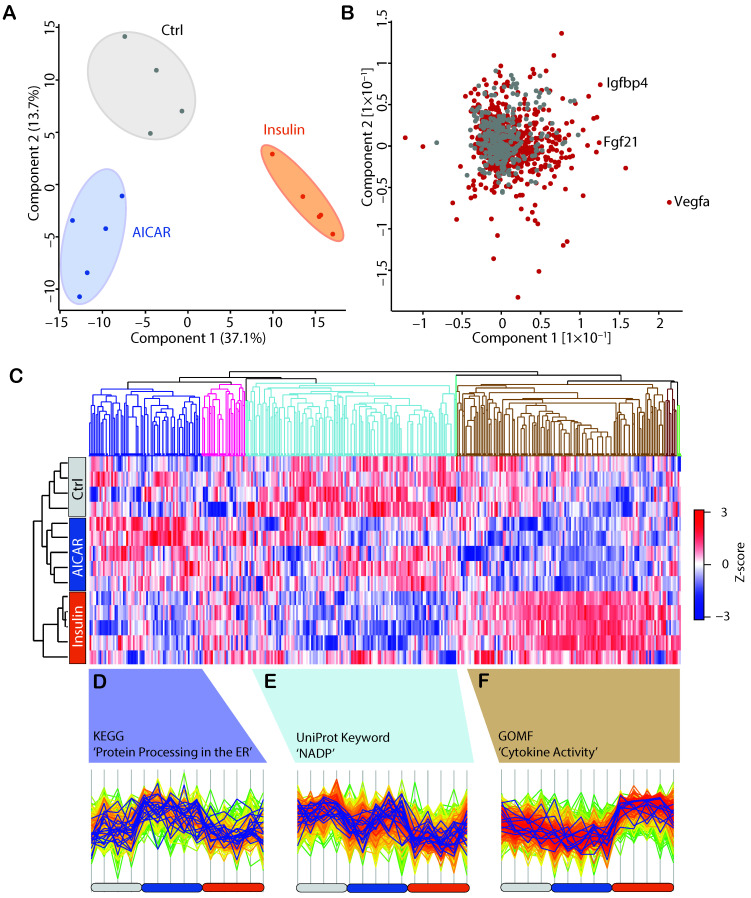
(**A**): Principal component analysis (PCA) of the Ctrl, AICAR- and insulin-stimulated secretome of C2C12 myotubes (*n* = 4–5). (**B**): PCA-loading analysis of the C2C12 secretome, with secreted proteins highlighted in red. (**C**): Z-score analysis of the C2C12 secretome reveals the enrichment of three clusters increased by AICAR (**D**), decreased by insulin (**E**) or increased by insulin (**F**).

Principal component analysis (PCA) revealed clear segregation between the three experimental conditions, suggesting that the composition of their secretomes is considerably distinct (Figure 3A). Secreted proteins are likely to drive this separation (Figure 3B). Among them are known secreted proteins including Fgf21, Vegfa and Igfbp4 (Figure 3B), which exert pleiotropic biological functions in skeletal muscle, as well as other peripheral tissues [46,47,48]. When analyzing the expression of all 1192 quantified proteins in the conditioned media, three differentially expressed clusters were revealed across the experimental conditions (Figure 3C). One cluster included proteins that increased following AICAR treatment (226 proteins; Figure 3D), and was enriched in proteins involved in protein processing in the ER (enrichment factor: 2.784, *p* < 0.0001, FDR = 0.0003). Of the proteins in this cluster, ~60% (167) were secreted proteins. Indeed, AMPK activation (at least with AICAR) can cause ER stress, and factors released secondary to AICAR treatment may possibly fine-tune or alleviate this process [49]. A second cluster revealed proteins decreased by insulin treatment (420 proteins, including 289 secreted proteins; Figure 3E), which contained proteins involved in pathways using NADP(H) as a cofactor (enrichment factor: 2.54, *p* < 0.0001, FDR = 0.00004). Redox cofactors do not readily migrate between cellular compartments, and proteins in this cluster may possibly respond to and communicate changes in redox balance to neighboring cells. Decades ago, researchers observed increases to reactive oxygen species (ROS) in response to the acute insulin stimulation of adipocytes, which is now recognized as a key mechanism for intact insulin signaling [50]. Accordingly, recent work demonstrates that active Akt may promote increases to NADP(H) expression, an enzyme that catalyzes the production of ROS [51]. Thus, factors secreted in response to insulin may possibly exist as part of a negative feedback loop to alleviate ROS production. A third cluster contained proteins that were increased by insulin (421 proteins, including 323 secreted proteins; Figure 3F), and this last cluster was enriched by proteins involved in cytokine activity, and included members such as Tgf-β1/2 (enrichment factor: 2.360, *p* < 0.001, FDR = 0.03). The Tgf-β family of proteins have emerged as beneficial coordinators of skeletal muscle metabolic responses to exercise [52], but have also been linked to the regulation of metabolism in several tissues, particularly in obesity and type 2 diabetes [53,54]. Given that skeletal muscle is a major site for insulin action, our findings may highlight the endocrine role of skeletal muscle-derived Tgf-β1/2 and raise the possibility that secretion may be altered in insulin resistant muscle. Altogether, more than two thirds of all proteins clustered with insulin treatment, suggesting that this hormone strongly regulates the skeletal muscle secretome.

### 4.4. Comparison of the AICAR- and Insulin-Stimulated Secretome

**Figure 4:**
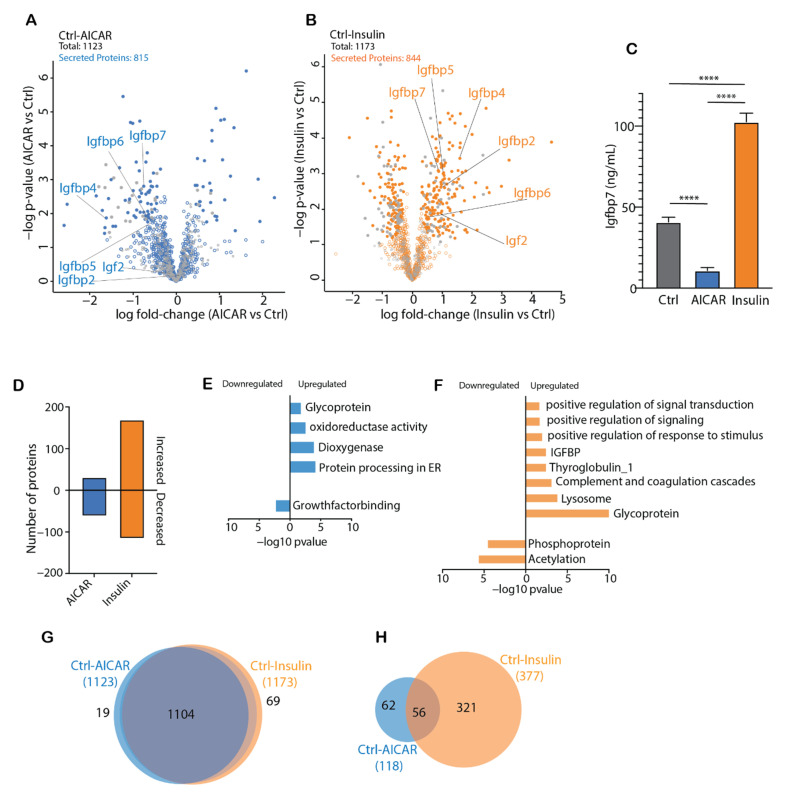
(**A**,**B**): Volcano plots including all proteins quantified in the conditioned media of control-AICAR (**A**) and control-insulin (**B**) paired comparisons. (**C**): Immunoassay measurement of IGFBP7 concentrations in the AICAR- and insulin-stimulated secretome (*n* = 6–8). (**D**): The number of secreted proteins that were either increased or decreased by AICAR and insulin treatment, compared to control. (**E**,**F**): Enrichment of biological pathways by AICAR (**E**) and insulin-stimulated (**F**) secreted proteins. (**G**): Venn diagrams highlighting the overlap of proteins quantified in the conditioned media of C2C12 myotubes treated with AICAR or insulin. (**H**): Venn diagrams highlighting the overlap of significantly regulated (versus control) proteins quantified in the conditioned media of C2C12 myotubes treated with AICAR or insulin. Data expressed as mean ± SEM. Immunoassay data were analyzed using a one-way ANOVA with Holm–Šídák post hoc test. **** *p* < 0.0001.

To discern the effects of AICAR and insulin in the regulation of protein secretion, we performed comparative analysis of AICAR and insulin-induced conditioned media secretomes (Figure 4A,B). Following AICAR treatment, 118 proteins were differentially regulated (35 upregulated and 83 downregulated), whereas insulin regulated 377 proteins (207 upregulated and 170 downregulated) (Figure 4A,B). Generally, proteomic analysis of conditioned media revealed that the signal peptide-containing family of Insulin-like Growth Factor Binding Proteins (Igfbp2, 4–7) showed opposing patterns of secretion following AICAR and insulin treatment (Figure 4A,B), and upon immunoassay validation of Igfbp7, this trend persisted (Figure 4A–C). Specifically, insulin increased, whereas AICAR decreased Igfbp7 concentrations in the conditioned media (Figure 4B). Insulin likely promotes the release of Igfbp proteins as part of a negative feedback loop to regulate Igf-signaling, which partly overlaps with cellular targets of insulin receptor activation [55,56]. In the case of Igfbp7, this protein does not bind Igf proteins with high affinity, but impairs Igf signaling at the receptor level [57].

In both paired comparisons, a similar number of secreted proteins were quantified, with 815 proteins in AICAR-stimulated and 844 proteins in insulin-stimulated myotubes (Figure 4A,B). However, the number of differentially expressed proteins was substantially different in the two comparisons and the directionality of protein abundance varied greatly (Figure 4D). AICAR decreased the abundance of proteins relating to growth factor binding (Figure 4E), whereas the abundance of secreted proteins with roles in oxidoreductase and dioxygenase activity, protein processing in the ER, as well as those annotated as glycoproteins was increased (Figure 4E). Despite its widespread use as an AMPK-activator in metabolic research, AICAR likely has many off-target and AMPK-independent effects [58,59,60]. In part, AICAR (or intermediary ZMP) may directly affect mitochondrial metabolism, and these targeted effects may relay signals for the release of distinct proteins that were observed to be involved in oxidoreductase activity [59,60,61]. Using enrichment analysis of differentially regulated proteins, phospho-proteins and proteins relating to post-translational acetylation were downregulated following insulin treatment, while other secreted proteins implicated in a variety of pathways were upregulated (Figure 4F). This included proteins associated with the positive regulation of signal transduction/signaling/response to stimuli, those involved in the complement pathway, lysosomal and glycoproteins, as well as proteins from the Igfbp family (Figure 4F). Thus, glycoproteins were increased by insulin, and reduced by AICAR, which could suggest that beyond regulating the composition of the secretome, insulin may also regulate components of the secretory machinery that influence protein glycosylation. Ultimately, this may help explain the greater protein dynamics in conditioned media following insulin treatment, since this post-translational modification is vital for the secretion of many proteins. Although there were many overlapping proteins in the AICAR- and insulin-stimulated conditioned media (compared to control), the majority of differentially altered secreted proteins were uniquely regulated by insulin (Figure 4H).

### 4.5. Conditioned Media from Insulin-Stimulated Myotubes Regulates Mitochondrial Respiration

**Figure 5:**
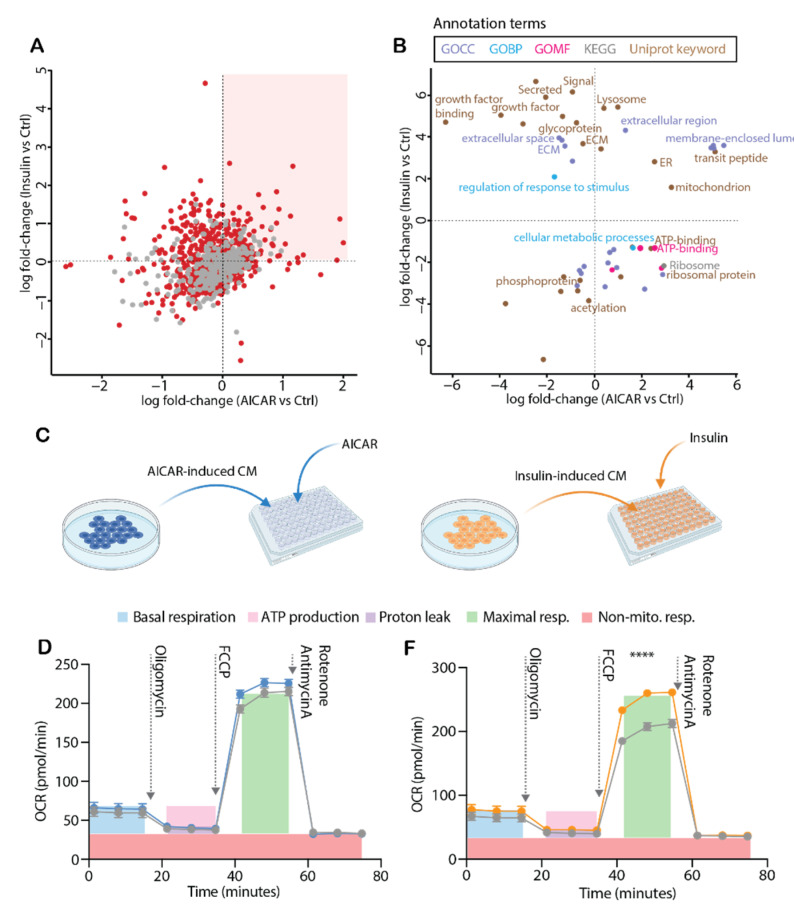

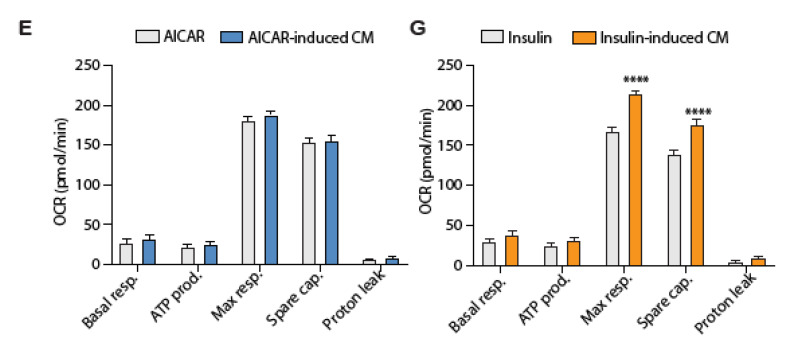
(**A**): Log fold-changes of proteins in the conditioned media for paired comparisons of Ctrl-AICAR and Ctrl-insulin, with secreted proteins highlighted in red. (**B**): 2D enrichment analyses of Ctrl-AICAR and Ctrl-insulin paired comparisons. (**C**): Schematic describing the collection of AICAR- (0.5 mM) and insulin- (10 nM) induced conditioned media for the measurement of mitochondrial respiration in another subset of C2C12 myotubes. (**D**,**F**): Oxygen consumption rate (OCR) following 4h treatment of myotubes with AICAR- or insulin-induced conditioned media (*n* = 27–30/group). AICAR- and insulin-induced conditioned media were compared against AICAR- or insulin-containing differentiation media alone (i.e., in the absence of cells and their secreted factors). (**E**,**G**): Mitochondrial respiratory parameters measured from OCR in (**D**,**F**). Data expressed as mean ± SEM from two independent experiments. (**D**,**F**): Data were analyzed using a two-way ANOVA with Holm-Šídák *post hoc* test, effect of treatment *p* = 0.0063. (**G**): Mixed-effects model with Holm–Šídák post hoc test, effect of treatment *p* < 0.0001. **** *p* < 0.0001.

When comparing the fold-change of AICAR and insulin-induced secretomes, we found insulin regulated a greater number of proteins (Figure 5A, top quadrants). Proteins that increased in both conditions are highlighted in the top right quadrant, and secreted proteins are shown in red (Figure 5A). Using a 2D enrichment analysis, we delved further into the cellular compartments, biological processes and molecular pathways/functions altered by the respective treatments (Figure 5B). Both AICAR and insulin increased mitochondrial-related proteins in the conditioned media, as well as proteins containing a transit peptide, which targets them to different organelles, including the mitochondria (Figure 5B). We then assessed whether insulin- and/or AICAR-stimulated secreted factors affected mitochondrial metabolism. Accordingly, we measured mitochondrial respiration of C2C12 myotubes following a four hour treatment with AICAR- or insulin-stimulated conditioned media (Figure 5C). AICAR-induced conditioned media did not affect mitochondrial respiration when compared to control media supplemented with AICAR alone (Figure 5D,E). In contrast, the insulin-induced conditioned media increased mitochondrial maximal respiration and spare capacity (Figure 5F,G), which may be driven by secreted proteins that were solely upregulated by insulin (Table S1). We cannot exclude the possibility that the insulin-mediated downregulation of proteins (116) may be of equal or greater importance in the observed fine-tuning of mitochondrial respiration. Future work is warranted to uncover the mechanisms by which insulin-induced secreted factors regulate mitochondrial metabolism, including whether treatment with insulin-induced conditioned media for four hours could alter the mitochondrial content of myotubes to increase mitochondrial flux. Accordingly, there is ongoing debate regarding the role of mitochondria dysfunction in the pathology of insulin resistance/type 2 diabetes [62]. Moreover, remodeling of mitochondrial content and function can occur in skeletal muscle after only one week ablation of insulin action in vivo [63], implicating a role for insulin signaling. In addition, the prolonged absence of insulin in human stem cells leads to significant reductions to mitochondrial respiratory parameters [64]. Future studies measuring tissue-secreted protein responses are warranted in these contexts. Regardless, the metabolic effects of insulin-induced myokines should be considered when interpreting the direct effects of insulin treatment, particularly in isolated experiments.

## 5. Conclusions

Since discovering the endocrine capacity of skeletal muscle, there has been increased motivation to identify and characterize myokines, which likely consist of peptides and proteins that drive the metabolic responses to exercise and pharmacological treatments. In this present study, we investigated secreted proteins from fully differentiated C2C12 myotubes in response to pathways that underpin nutrient uptake, energy metabolism and therefore, metabolic disease. Apart from identifying several well-known proteins (e.g., Tgf-β, Vegfa, and Fgf21) with effects on energy metabolism, we also provide a resource for researchers concerned with the secondary biological implications of insulin or AMPK action. Indeed, the ability of metabolic hormones and compounds to regulate cellular secretory action may confound the direct interpretation of their physiological effects. Accordingly, the physiological adaptations following AICAR administration in vivo are starting to be linked to secreted proteins [15]. Although the regulation of insulin-induced myokines remains largely unexplored, scenarios of insulin resistance alter the secretory response of skeletal muscle [11,44]. This is particularly interesting since insulin potently and differentially regulates the skeletal muscle secretome, at least when compared to energetic stressors (i.e., AMPK activation). Notably, members of the Fgf and Igfbp family were upregulated by insulin treatment. These myokines, among others, represent important avenues for future research, particularly in the context of insulin resistance. These, or other factor(s) present in the conditioned media from insulin- but not AICAR-stimulated myotubes may contribute to the observed increase in mitochondrial respiration. However, additional work will need to dissect the mechanisms driving this effect. Collectively, our findings highlight the utility of differentiated C2C12 myotubes as a viable and flexible tool for studying skeletal muscle secreted proteins. Moving forward, how different inputs beyond exercise training and muscle contractions directly regulate myokine release is important to ascertain in order to better contextualize the impact on physiology and metabolic research. While many proteins may become annotated as secreted in the future, notably even in the absence of experimental treatment, cultured cells secrete an astonishing number of proteins into the surrounding media. This may constitute a major consideration in the experimental design and interpretation of cell culture studies.

## Data Availability

The mass spectrometry proteomics data have been deposited to the ProteomeXchange Consortium (SCR_004055) via the PRIDE partner repository. The dataset identifier is PXD025687.

## Supplementary Materials

The following are available online at https://www.mdpi.com/2227-7382/9/3/37/s1, Table S1. Secretome from AICAR- and insulin-treated C2C12 myotubes. Table S2. Enrichment analysis for secreted proteins and differentially expressed proteins. Table S3. Proteome of AICAR- and insulin-treated C2C12 myotubes.
